# Supplementary material for: Drug Combinations to Prevent Antimicrobial Resistance: Various Correlations and Laws, and Their Verifications, Thus Proposing Some Principles and a Preliminary Scheme
Source: Antibiotics (Basel). 2022 Sep 20;11(10):1279. doi: 10.3390/antibiotics11101279 (PMC9598766; doi:10.3390/antibiotics11101279)
Supplement: Supplementary file 1 [file antibiotics-11-01279-s001.zip › antibiotics-1878949-supplementary.pdf]

Supplementary file

Drug combinations to prevent antimicrobial resistance: various correlations and laws, and their verifications, thus proposing some principles and a preliminary scheme

Houqin Yi [1,2], Ganjun Yuan [1,2,*], Shimin Li [1,2], Xuejie Xu [1], Yingying Guan [1,2], Li Zhang [2] and Yu Yan[2]

[1] Biotechnological Engineering Center for Pharmaceutical Research and Development, Jiangxi Agricultural University, Nanchang 330045, China
[2] Laboratory of Natural Medicine and Microbiological Drug, College of Bioscience and Bioengineering, Jiangxi Agricultural University, Nanchang 330045, China

* Correspondence: gyuan@jxau.edu.cn (G.Y.); Tel.: +86-0791-83813459

Table S1. The correlation between the ratio value of a_1/a_2 and that of MIC_2/MIC_1. (n = 9)

Table S2. The correlation between the ratio value of b_1/b_2 and that of MIC_2/MIC_1. (n = 9)

Table S3. The rule $b_1+b_2 = -1$ and the correlation between the ratio of $b_{larger}/b_{smaller}$ (x) and $MPC_{larger}/MPC_{smaller}$ (y) for the equations $y = a_1x^{b1}$ and $y = a_2x^{b2}$ of two agents in a drug combination. (n = 9)

Table S4. The correlation between the ratio of $a_{larger}/a_{smaller}$ (x) and $MPC_{larger}/MPC_{smaller}$ (y) for the equations $y = a_1\ln(x)+b_1$ and $y = a_2\ln(x)+b_2$ of two agents in a drug combination. (n = 9)

Table S5 The correlations between the tested MPSI and the calculated MPSI, the tested MPSI and the tested MPSI (1:1), and the tested MPSI (1:1) and the calculated MPSI (1:1). (n = 9)

Table S6. The MIC and corresponding MPC, of various antimicrobial agents reported in seventeen papers.

Table S7. Evaluation to the prediction of the MPC from the MIC of an antimicrobial agent.

Table S1. The correlation between the ratio value of a_1/a_2 and that of MIC_2/MIC_1. (n = 9) [a]

a_1	a_2	a_1/a_2 (x)	MIC_1 (μg/mL)	MIC_2 (μg/mL)	MIC_2/MIC_1 (y)	Regression equation [b]
0.3613	0.1838	1.97	0.13	0.25	1.92	
0.3392	0.3669	0.92	0.13	0.13	1.00	
0.0124	3.058	0.0041	32	0.13	0.0041	
2.6565	2.6561	1.00	1	1	1.00	$y = 0.9602x$ ($x > 0$), $r = 0.9993$
1.7752	1.7769	1.00	2	2	1.00	
3.6279	0.7627	4.76	0.5	2	4.00	$y = 0.9932x$ ($x \geq 1$), $r = 0.9998$ [c]
4.5965	0.0716	64.20	1	64	64.00	
2.5407	0.1587	16.01	2	32	16.00	
10.24	0.1483	69.05	0.5	32	64.00	

[a]: The values of a_1 and a_2 were obtained from the equations of Type I in Table 1, and the MIC_1 and MIC_2 were obtained from Tables 1 and 2 of our previous paper [24].

[b]: The regression equations were established using Microsoft Excel software and setting the intercept to zero; r, correlation coefficient.

[c]: The reciprocals of a_1/a_2 were taken for the equation establishment when the calculated values of a_1/a_2 are less than 1, and correspondingly the reciprocals of the MIC_2/MIC_1 values were also taken.

Table S2. The correlation between the ratio value of b_1/b_2 and that of MIC_2/MIC_1. (n = 9) [a]

b_1	b_2	b_1/b_2 (x)	MIC_1 (μg/mL)	MIC_2 (μg/mL)	MIC_2/MIC_1 (y)	Regression equation [b]
0.456	0.2629	1.73	0.13	0.25	1.92	
0.3729	0.5385	0.69	0.13	0.13	1.00	
0.0251	3.0703	0.0082	32	0.13	0.0041	
3.5271	3.2771	1.08	1	1	1.00	
2.1907	2.3864	0.92	2	2	1.00	$y = 1.9721x$ ($x > 0$), $r = 0.9910$
4.4196	1.0457	4.23	0.5	2	4.00	
4.8057	0.1719	27.96	1	64	64.00	
2.8193	0.2629	10.72	2	32	16.00	
10.541	0.3019	34.92	0.5	32	64.00	

[a]: The values of b_1 and b_2 were obtained from the equations of Type II in Table 1, and the MIC_1 and MIC_2 were obtained from Tables 1 and 2 of our previous paper [24].

[b]: The regression equations were established using Microsoft Excel software and setting the intercept to zero; r, correlation coefficient.

[c]: The reciprocals of b_1/b_2 were taken for the equation establishment when the calculated values of a_1/a_2 are less than 1, and correspondingly the reciprocals of the MIC_2/MIC_1 values were also taken.

Table S3. The rule $b_1+b_2 = -1$ and the correlation between the ratio of $b_{larger}/b_{smaller}$ (x) and $MPC_{larger}/MPC_{smaller}$ (y) for the equations $y = a_1x^{b1}$ and $y = a_2x^{b2}$ of two agents in a drug combination. (n = 9) [a]

b_1	b_2	b_1+b_2	$b_{larger}/b_{smaller}$ (x)	MPC_1 (μM/L)	MPC_2 (μM/L)	$MPC_{larger}/MPC_{smaller}$ (y)	Regression equation [b]
−0.487	−0.618	−1.11	1.269	0.251	5.761	22.952	
−0.519	−0.481	−1.00	1.079	10.627	70.836	6.666	
0.046	−1.049	−1.00	−22.804	10.627	7241.130	681.390	
−0.318	−0.678	−1.00	2.132	0.191	4.500	23.560	$y = 28.831x - 27.831$
−0.463	−0.537	−1.00	1.160	11.041	53.127	4.812	$r = 0.9985$ ($n = 7$)
−0.278	−0.722	−1.00	2.597	11.041	7241.130	655.840	
−0.925	−0.071	−1.00	13.028	305.818	0.878	348.312	
−0.569	−0.619	−1.19	1.088	10.351	22.136	2.139	
−0.129	−0.93	−1.06	7.209	10.351	1853.729	179.087	

[a]: The values of b_1 and b_2 were obtained from the equations of Type I in Table 1, and the MPC_1 and MPC_2 were calculated from the corresponding data in Tables 1 and 2 of our previous paper [24].

[b]: The regression equation was established using Microsoft Excel software and setting the intercept to −27.831; r, correlation coefficient; the equation was established using seven data pairs (n =7) as (2.597, 655.840) strayed from the group, and the value of b_2/b_1 (−22.804) was negative since the difference between the MPCs of two agents was very larger (this was also supported that the larger the difference between the MPC values of two agents in a drug combination, the more obvious and larger the difference between the b values of both two equations).

Table S4. The correlation between the ratio of $a_{larger}/a_{smaller}$ (x) and $MPC_{larger}/MPC_{smaller}$ (y) for the equations $y = a_1\ln(x) + b_1$ and $y = a_2\ln(x)+b_2$ of two agents in a drug combination. (n = 9) [a]

a_1	a_2	$a_{larger}/a_{smaller}$ (x)	MPC_1 (μM/L)	MPC_2 (μM/L)	$MPC_{larger}/MPC_{smaller}$ (y)	Regression equation [b]
−0.161	−0.126	1.278	0.251	5.761	22.952	
−1.509	−1.246	1.211	10.627	70.836	6.666	
0.2592	−0.122	−2.125	10.627	7241.130	681.390	
−0.108	−0.289	0.374	0.191	4.500	23.560	
−0.929	−1.217	0.763	11.041	53.127	4.812	$y = 29.956x - 28.956$
−0.747	−0.156	4.788	11.041	7241.130	655.840	$r = 0.9521$ ($n = 7$)
−0.018	−0.203	0.089	305.818	0.878	348.312	
−1.951	−0.463	4.214	10.351	22.136	2.139	
−1.464	−0.212	6.906	10.351	1853.729	179.087	

[a]: The values of a_1 and a_2 were obtained from the equations of Type II in Table 1, and the MPC_1 and MPC_2 were calculated from the corresponding data in Tables 1 and 2 of our previous paper [24].

[b]: The regression equation was established using Microsoft Excel software and setting the intercept to −29.956; r, correlation coefficient; the equation was established using seven data pairs (n =7) as (4.788, 655.840) strayed from the group, and the value of a_2/a_1 (−2.125) was negative since the difference between the MPCs of two agents was very larger (this was also supported that the larger the difference between the MPC values of two agents in a drug combination, the more obvious and larger the difference between the a values of both two equations).

Table S5 The correlations between the tested MPSI and the calculated MPSI, the tested MPSI and the tested MPSI (1:1), and the tested MPSI (1:1) and the calculated MPSI (1:1). (n = 9) [a]

MRSA isolates	Combinations [b] (A/B)	MPSIs [c]			MPSIs (1:1) [c]		
		Tested	Calculated [d]	Calculated [e]	Tested	Calculated [d]	Calculated [e]
01	RM/DC	0.082	0.061	0.07	0.082	0.080	0.557
	VM/OX	0.470	0.651	1.763	0.602	0.601	0.929
	VM/FF	0.002	0.002	−0.015	0.015	0.015	0.036
02	RM/DC	0.040	0.011	−0.421	0.080	0.087	1.444
	VM/OX	0.554	0.715	−0.557	0.833	0.834	1.089
	VM/FF	0.008	0.006	−0.049	0.016	0.016	0.093
03	RM/DC	3094.505 [f]	3908.6 [f]	215.3	656.410 [f]	657.6 [f]	122.3 [e]
	VM/OX	0.778	1.421	0.229	1.875 [f]	1.577 [f]	0.237
	VM/FF	0.008	0.010	−0.019	0.059	0.054	0.029
Correlations between the tested MPSI (x) and the calculated MPSI (y)			$y = 1.5884x$ $r = 0.9867$ **	Not applicable			
Correlations between the tested MPSI (1:1) (x) and the calculated MPSI (1:1) (y)						$y = 1.0398x$ $r = 0.9972$ **	$y = 0.4523x$ $r = 0.4562$
Correlations between the tested MPSI (x) and the tested MPSI (1:1) (y)				$y = 1.9392x$ $r = 0.9672$ **			

[a]: r, correlation coefficient; using r-test, the very significant difference ($P < 0.01$) was marked as **, and the critical value of $r_{0.995}(7)$ is equal to 0.798 when the significant levels α were set as 0.01.

[b]: RM, roxithromycin; DC, doxycycline; VM, vancomycin; FF, fosfomycin; OX, ofloxacin.

[c]: These tested MPSIs (also shown in Table 3) and MPSIs (1:1) were calculated from Tables 1 and 2 in our previous publication [24], according to Formula (2).

[d]: The calculated MPSIs (1:1) were calculated from the corresponding regression equations $y = ax^b$ in Table 1 when $x = 1$.

[e]: The calculated MPSIs (1:1) were calculated from the corresponding regression equations $y = a\ln(x) + b$ in Table 1 when $x = 1$.

[f]: The reciprocals were taken when calculated, except for the correlation between the tested MPSI and the tested MPSI (1:1).

Table S6 The MIC and corresponding MPC, of various antimicrobial agents reported in seventeen papers [11,12,23,24,32-41]

Data pairs		Data pairs		Data pairs		Data pairs		Data pairs		Data pairs		Data pairs	
MIC	MPC	MIC	MPC	MIC	MPC	MIC	MPC	MIC	MPC	MIC	MPC	MIC	MPC
**R12**		0.125	4	1.80	7.00	1	64	1	8	32	256	0.25	8
0.5	4	1	16	**R11**		1	64	**R37**		R39		1	128
1	8	1	8	1	8	1	64	0.25	4	0.09	0.34	0.5	64
1	4	0.5	8	2	16	**R34**		0.0625	0.5	0.12	0.39	0.5	4
1	16	0.25	4	1	32	2	7.2	0.125	2	0.125	0.5	0.031	1
1	8	0.5	8	1	16	2	8	0.6	8	0.125	0.5	0.5	16
2	8	1	8	0.5	16	2	10.2	0.03	0.4	0.25	1	4	256
**R32**		1	8	2	16	4	51.2	0.04	0.9	0.25	8	0.5	16
1	16	0.5	4	0.5	16	4	57.6	15	2000	1	2	0.5	8
4	32	0.25	8	1	16	0.5	14.4	230	2800	0.5	2	0.25	4
4	64	**R24**		2	32	**R35**		6	80	1	4	1	16
2	32	0.13	0.21	**R33**		1024	16384	0.25	3	1	32	0.5	16
4	64	1	15.4	1	32	32	256	0.12	0.38	**R40**		0.5	16
2	32	0.13	0.16	1	32	64	256	0.5	32	0.25	4	0.031	1
4	64	2	16	0.5	32	16	64	0.014	0.22	0.031	1	0.125	32
1	16	32	256	0.5	16	16	64	3	17	0.5	32	1	256
4	64	0.5	15	0.5	32	16	16	0.24	1.2	4	512	0.125	16
4	32	0.25	2.56	0.5	16	4	64	**R38**		0.5	16	1	32
1	32	1	25.6	0.5	16	1	16	64	2048	1	64	0.125	4
2	64	64	1000	1	32	2	64	64	2048	0.125	8	0.5	32
2	64	0.13	2	0.5	32	2	4	64	2048	0.5	128	0.25	4
2	32	2	19.2	0.5	32	1	8	64	2048	1	64	**R41**	
1	32	32	1000	0.25	8	0.5	4	64	2048	0.25	4	0.125	4
2	64	0.13	0.39	0.25	4	0.25	8	2	16	0.063	1	0.5	16
2	32	2	8	0.25	8	0.25	2	4	32	1	32	1.00	8
2	64	32	256	0.125	4	**R36**		4	32	4	512	2.00	16
2	32	**R23**		0.5	16	0.25	2	8	64	0.25	16	0.5	64
0.5	64	0.20	3.40	0.5	64	0.5	4	64	256	0.5	64		

[a]: R11, R12, R23, R24, R32 to R41 were respectively references 11, 12, 23, 24, 32 to 41. The information involving antimicrobial agents and pathogenic bacteria were not shown since whether the correlation between the MIC and MPC of an antimicrobial agent is established should not relate to a specific antimicrobial agent against a specific pathogenic bacterium.

Table S7. Evaluation of the MPC predicted from the MIC of an antimicrobial agent.[a]

MIC	MPC Tested	MPC predicted [b]	MPC predicted [c]	Evaluation [d]	MIC	MPC tested	MPC predicted [b]	MPC predicted [c]	Evaluation [d]
**R42**					**R44**				
1	32	25.44	16.03	C	0.063	0.5	1.61	1.01	C
2	64	50.75	32.05	C	0.031	0.5	0.79	0.50	C
1	32	25.44	16.03	C	0.063	0.25	1.61	1.01	A
0.5	32	12.74	8.01	A	0.031	0.25	0.79	0.50	C
1	32	25.44	16.03	C	0.008	0.016	0.20	0.13	U
1	16	25.44	16.03	C	0.125	1	3.19	2.00	C
1	32	25.44	16.03	C	0.063	0.25	1.61	1.01	A
1	16	25.44	16.03	C	0.063	0.5	1.61	1.01	C
1	32	25.44	16.03	C	0.031	0.25	0.79	0.50	C
0.5	16	12.74	8.01	C	0.008	0.016	0.20	0.13	U
**R43**					0.125	2	3.19	2.00	C
4	>64	100.93	64.10	C	0.031	0.25	0.79	0.50	C
0.063	0.5	1.61	1.01	C	0.063	0.25	1.61	1.01	A
0.031	0.25	0.79	0.50	C	0.016	0.125	0.41	0.26	A
0.063	0.5	1.61	1.01	C	0.008	0.031	0.20	0.13	U
0.063	0.5	1.61	1.01	C	**R45**				
8	>64	199.61	128.20	C	0.125	1	3.19	2.00	C
0.13	2	3.32	2.08	C	0.16	1.024	4.08	2.56	A
0.25	2	6.37	4.01	C	0.4	10.24	10.19	6.41	C
0.25	2	6.37	4.01	C	0.5	4	12.74	8.01	C
0.5	2	12.74	8.01	A	0.016	0.016	0.41	0.26	U
**R46**					**R46**				
0.25	4	6.37	4.01	C	2	16	50.75	32.05	A
2	32	50.75	32.05	C	0.5	8	12.74	8.01	C
0.25	4	6.37	4.01	C	2.00	16.00	50.75	32.05	C

C: 73.9%; A:17.4%; A+C: 91.3%; U:8.7%

[a]: R42 to R46 were respectively references 42 to 46.

[b]: The MPCs were calculated from equation (8) $y = 0.00006x^3 - 0.07104x^2 + 25.5154x$.

[c]: The MPCs were calculated from equation (7) $y = 16.025x$.

[d]: Comparison to the reported MPC, C, Complete coincidence; A, Acceptable; U, Unacceptable. Considering that many factors, such as determination method, concentration of bacterial suspension, and test medium used, may influence on the determination of MIC, the results reported would fluctuate within a reasonable range of the actual values. Therefore, the predicted MPCs ranged from 1/4 × to 4 × the determined one were acceptable (marked as A), especially those ranged from 1/2 × to 2 × the determined one, were considered as complete coincidence (marked as C) since the MICs were generally determined by double dilution method and there is a positive correlation between the MIC and MPC as equation (3). Simultaneously, those falling within a specific MIC range (such as > 64) were also regarded as complete coincidence (marked as C). Otherwise, those were unacceptable (marked as U).a
